# Phase 1b study of intravenous coxsackievirus A21 (V937) and ipilimumab for patients with metastatic uveal melanoma

**DOI:** 10.1007/s00432-022-04510-3

**Published:** 2023-01-18

**Authors:** Jose Lutzky, Ryan J. Sullivan, Justine V. Cohen, Yixin Ren, Anlong Li, Rizwan Haq

**Affiliations:** 1 Sylvester Comprehensive Cancer Center, University of Miami Miller School of Medicine, 1475 NW 12th Ave, Miami, FL 33136 USA; 2 Massachusetts General Hospital, Boston, MA USA; 3 Merck & Co., Inc., Rahway, NJ USA; 4 Department of Medical Oncology, Dana-Farber Cancer Institute, Harvard Medical School, Boston, MA USA

**Keywords:** Oncolytic virotherapy, Oncolytic viruses, Immunotherapy, Melanoma

## Abstract

**Purpose:**

No standard of care therapy exists for patients with metastatic uveal melanoma who are not HLA-A2:01 positive. The phase 1b, open-label CLEVER study (NCT03408587) evaluated V937 in combination with ipilimumab in patients with uveal melanoma.

**Methods:**

Adults with advanced uveal melanoma and liver metastases received up to 8 cycles of intravenous V937 (1 × 10^9^ TCID_50_ per infusion; infusions on days 1, 3, 5, and 8 [cycle 1], then every 3 weeks [Q3W] thereafter [cycles 2–8]) and 4 cycles of intravenous ipilimumab 3 mg/kg Q3W (beginning at cycle 1 day 8). The primary endpoint was safety. Secondary endpoints included objective response rate and progression-free survival (PFS) per immune-related Response Evaluation Criteria in Solid Tumors (irRECIST).

**Results:**

Eleven patients were enrolled (median age, 65.0 years) and received a median of 6 injections of V937 and 3.5 infusions of ipilimumab. The best overall response was stable disease in 3 patients and progressive disease in 8 patients. All patients exhibited progression per irRECIST, with a 9% irPFS rate at week 26. Ten patients had treatment-related AEs, the most frequent of which were diarrhea (55%), fatigue (45%), and myalgia (36%). Two grade 3 AEs (diarrhea, *n* = 2) were considered related to ipilimumab; neither was related to V937.

**Conclusion:**

Although the combination of V937 with ipilimumab had a manageable safety profile, meaningful clinical benefit was not observed in patients with uveal melanoma and liver metastases.

**Trial registration:**

ClinicalTrials.gov, NCT03408587 (January 24, 2018).

## Introduction

Antitumor activity of oncolytic viruses is thought to occur through both oncolysis and induction of a systemic antitumor immune response (Kaufman et al. 2015). In 2015, the first commercially available oncolytic virus, talimogene laherparepvec (T-VEC), was approved by the US Food and Drug Administration to treat unresectable, recurrent melanoma (Andtbacka et al. 2015; Imlygic® 2021). Coxsackievirus A21 (CVA21) is a naturally occurring virus that infects cells in the respiratory tract, inducing mild upper respiratory symptoms (Spickard et al. 1963). CVA21 exploits the presence of intercellular adhesion molecule-1 (ICAM-1) to bind and infect the host cell (Au et al. 2007; Shafren et al. 1997), and melanoma has been identified as a cancer type with high ICAM-1 levels relative to normal cells (Shafren et al. 2004). Three clinical studies have evaluated V937, an unmodified bio-selected strain of CVA21, in patients with cutaneous melanoma. In the phase 2 CALM study (NCT01227551, NCT01636882 [extension]), intratumoral administration of V937 to 57 patients with unresectable, advanced cutaneous melanoma was associated with a 6-month progression-free survival (PFS) rate of 39% and objective response rate (ORR) of 39% (Andtbacka et al. 2021). In the phase 1b MITCI study (NCT02307149), 50 patients with unresectable, advanced melanoma received intratumoral injections of V937 and intravenous ipilimumab, achieving an ORR of 30%, median PFS of 6.2 months, and median overall survival (OS) of 45.1 months; responses were robust and higher than anticipated with ipilimumab monotherapy, including in patients who had received prior anti–PD-1 therapy (Bifulco et al. 2021). No grade 5 treatment-related adverse events (AEs) occurred; toxicities were manageable and consistent with those expected for the individual treatment components. In the phase 1 STORM study (NCT02043665), intravenous infusion of V937 in combination with pembrolizumab was generally well tolerated and showed evidence of viral replication in tumor biopsies from patients with melanoma (Pandha et al. 2017).

Uveal melanoma has also been identified as a cancer type with high ICAM-1 expression, particularly in the metastatic setting (Anastassiou et al. 2000). Uveal melanoma accounts for 85% of primary cancers of the eye and 5% of melanomas (Carvajal et al. 2017; Chang et al. 1998; Chattopadhyay et al. 2016). The underlying biology of uveal melanoma differs from that of cutaneous melanoma. Cutaneous melanoma occurs in melanocytes in the basal layer of the epidermis, whereas uveal melanoma can occur anywhere in the uveal tract, including the choroid, iris, or ciliary body (Carvajal et al. 2017). Uveal melanoma tumors have fewer mutations and a different mutational spectrum than cutaneous melanoma, which may limit the efficacy of immunotherapy in this setting (Chattopadhyay et al. 2016; Snyder et al. 2014). Most patients eventually develop distant metastasis, for which no standard of care currently exists (Carvajal et al. 2017; Kujala et al. 2003). Patients with metastatic uveal melanoma have often been treated with systemic therapies that are approved for cutaneous melanoma, such as chemotherapy or checkpoint inhibitors (Carvajal et al. 2014). The National Comprehensive Cancer Network recommends both approaches despite limited response rates (ORRs of 0%–5% have been reported for chemotherapy and 0%–18% for checkpoint inhibitors) (Algazi et al. 2016; Carvajal et al. 2014; Chattopadhyay et al. 2016; Maio et al. 2013; Najjar et al. 2020; Namikawa et al. 2020; National Comprehensive Cancer Network 2021; Pelster et al. 2021; Rossi et al. 2019; Zimmer et al. 2015). In a recent phase 3 study (NCT03070392), the T-cell–redirecting bispecific fusion protein tebentafusp was associated with statistically significant improvements in 1-year OS (73%) compared with pembrolizumab, ipilimumab, or dacarbazine monotherapy (59%) for HLA-A*02:01–positive patients with metastatic uveal melanoma (Nathan et al. 2021). On January 25, 2022, the Food and Drug Administration approved tebentafusp-tebn (Kimmtrak^®^, Immunocore Limited) for HLA-A*02:01–positive adult patients with unresectable or metastatic uveal melanoma. HLA-A02:01–positive patients comprise approximately 45% of the metastatic uveal melanoma patient population (Nathan et al. 2021). Given the limited treatment options for metastatic uveal melanoma, a need exists for treatments that provide improved outcomes for patients with this disease. CLEVER (NCT03408587) was an open-label, phase 1b, multicenter study that evaluated the safety and clinical activity of intravenous V937 in combination with ipilimumab in patients with metastatic uveal melanoma. This combination was chosen for investigation based primarily on the favorable efficacy and safety results from the previously mentioned MITCI study and the complementary mechanisms of action of the treatments.

## Methods

### Patients

Eligible patients were ≥ 18 years of age and had a histologically or cytologically confirmed diagnosis of uveal melanoma per Response Evaluation Criteria in Solid Tumors (RECIST) version 1.1 and metastases to the liver, with an estimated tumor volume in the liver less than one-third of the total liver volume (based on computed tomography [CT] or magnetic resonance imaging [MRI]) and no single metastatic lesion more than 8 cm in the longest diameter. Additional eligibility criteria included an Eastern Cooperative Oncology Group (ECOG) performance status of 0 or 1, adequate organ function, and life expectancy > 12 weeks. Patients who received prior treatment were required to have progression per RECIST version 1.1 during or after the last treatment. Prior treatment with an immune checkpoint inhibitor was permitted, following a 6-week washout period. Patients were excluded if they were candidates for surgery or locoregional treatment with curative intent; had active central nervous system metastases or any other known malignancy (other than squamous cell or basal cell carcinoma treated with potentially curative therapy or in situ cervical cancer) that was progressing or required active treatment; were receiving systemic steroid therapy at > 10 mg prednisone or equivalent; had active autoimmune disease, active colitis or previous immune-mediated colitis that has not resolved to grade ≤ 1, hepatitis B or C, or grade > 2 ascites; had a history of human immunodeficiency virus; had received chemotherapy, targeted small-molecule therapy, radiation therapy, hormonal treatment, or immunotherapy within 21 days before the first dose of study drug or had any residual grade > 1 toxic effects (or grade > 2 alopecia or neuropathy) from the most recent therapy; or had any other concurrent, uncontrolled illness, condition, therapy, or laboratory abnormality that might interfere with the patient’s participation or confound study results.

All patients provided written informed consent to participate. The study was conducted in accordance with the Declaration of Helsinki, the International Council for Harmonisation Good Clinical Practice guidelines, and all applicable local and national laws. The institutional review board at each study site approved the protocol, all protocol amendments, and informed consent forms before the study began.

### Study design and treatment

**Fig. 1:**
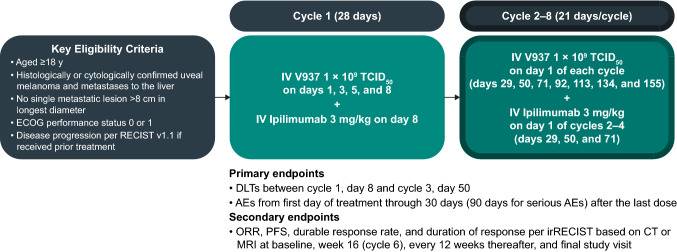
Study schema and endpoints. *AEs* adverse events, *CT* computed tomography, *DLTs* dose-limiting toxicities, *ECOG* Eastern Cooperative Oncology Group, *IV* intravenous, *MRI* magnetic resonance imaging, *ORR* objective response rate, *PFS* progression-free survival, *RECIST* Response Evaluation Criteria in Solid Tumors, *TCID*_*50*_ 50% tissue culture infectious dose

Patients received up to 8 cycles of intravenous V937 at 1 × 10^9^ 50% tissue culture infectious dose (TCID_50_) per infusion (Fig. 1). The first cycle was a 28-day cycle with infusions given on days 1, 3, 5, and 8. Subsequent cycles were 21 days in length with infusions given on day 1. Up to four 21-day cycles of intravenous ipilimumab 3 mg/kg were administered beginning on day 8 (ie, days 8, 29, 50, and 71). On days when both V937 and ipilimumab were administered, V937 was infused first.

The study was planned to enroll 6 patients for a 6-week dose-limiting toxicity (DLT) observation period that began with the commencement of ipilimumab therapy on day 8 and ended on day 50. Patients enrolled in the DLT observation period received study treatment per the protocol schedule. DLTs included any of the following considered related to V937 or ipilimumab: grade ≥ 4 hematologic toxicity lasting ≥ 7 days and/or complicated by infection; grade ≥ 4 thrombocytopenia; grade ≥ 3 febrile neutropenia; and any grade ≥ 3 nonhematologic toxicity (with the exception of grade 3 fatigue lasting < 7 days) that was considered a DLT and not a standard ipilimumab toxicity. If at least 2 patients experienced a DLT during the observation period, then V937 dosing would be stopped and the sponsor and investigator would determine whether to continue at a lower V937 dose. If no more than 1 patient experienced a DLT during the observation period, up to 4 additional patients were enrolled (ie, 10 total patients). If V937 treatment was discontinued due to toxicity, ipilimumab treatment could continue. Toxicities related to ipilimumab were managed according to the ipilimumab prescribing information (Yervoy® 2015). V937 dosing could continue if ipilimumab treatment was stopped.

### Assessments

AEs were assessed from the first day of treatment through 30 days after the last dose (90 days for serious AEs) and were graded using the National Cancer Institute Common Terminology Criteria for Adverse Events, version 5.0. Efficacy was assessed by the investigator using immune-related RECIST (irRECIST) (Bohnsack et al. 2014) based on CT or MRI performed at baseline, week 16 (ie, cycle 6), every 12 weeks thereafter, and at the final study visit.

### Endpoints

The primary endpoint was the occurrence of DLTs, AEs, and changes from baseline in other safety measures, such as laboratory values. Secondary endpoints included evaluations of best response (eg, ORR, defined as partial [PR] or complete response [CR]) and PFS per irRECIST (defined as the time from beginning of treatment to progressive disease [PD] or death, whichever occurred first), durable response rate (defined as the percentage of patients with CR or PR lasting ≥ 26 weeks), and duration of response (DOR; time from first evidence of PR or CR to confirmation of PD).

### Statistical analysis

Progression-free survival and DOR were analyzed using the Kaplan–Meier method. All other data were analyzed using summary statistics.

## Results

### Study population

Eighteen patients were screened and 11 were included in the study. Reasons for screen failure were no measurable disease in the liver (*n* = 2), withdrawal of consent (*n* = 2), no measurable disease (*n* = 1), enrollment on hold (*n* = 1), and active central nervous system metastasis (*n* = 1). Median time from the start of study treatment to the data cutoff date (June 21, 2019) was 10.3 months (range, 5.2−15.3 months). Among the enrolled patients, the median age was 65.0 years (range, 54–70 years), 6 patients were female, and 8 had a baseline ECOG performance status of 0 (Table 1).

**Table 1:** Demographics and baseline disease characteristics

	Patients (*N* = 11)
Age, median (range), years	65.0 (54–70)
Sex, *n* (%)	
Men	5 (45)
Women	6 (55)
ECOG performance status, *n* (%)	
0	8 (73)
1	3 (27)
Histologic diagnosis of uveal melanoma, *n* (%)	
Mixed cell	1 (9)
Mixed cell type	2 (18)
Spindle B-cell melanoma	1 (9)
Spindle cell neoplasm	1 (9)
Spindle cell type	1 (9)
Unknown	5 (45)
Time since initial diagnosis of primary uveal melanoma to study treatment, median (range), months	52.7 (18.8–289.6)
Time since last recurrence to study treatment, median (range), months	2.04 (0.4–3.7)
Prior radiotherapy, *n* (%)	10 (91)
Prior surgery, *n* (%)	9 (82)
Received prior systemic treatment, *n* (%)	6 (55)
Number of prior systemic treatments,^a^ *n* (%)	
0	5 (46)
1	2 (18)
2	1 (9)
3	2 (18)
6	1 (9)
Prior systemic treatment type, *n* (%)	
Adjuvant	3 (27)
Locally advanced/metastatic	5 (46)
Primary treatment	1 (9)

*ECOG* Eastern Cooperative Oncology Group

^a^Includes adjuvant therapy and therapy for metastatic disease

Ten patients (91%) discontinued the study due to symptomatic disease progression (i.e., comprising clinical progression and radiological progression) and 1 patient (9%) remained on treatment. Patients received a median of 6 injections (range, 3–11 injections) of V937 and 3.5 infusions (range 1–4 infusions) of ipilimumab. The median total dose administered was 6 × 10^9^ TCID_50_ (range 3–11 × 10^9^ TCID_50_) for V937.

### Efficacy outcomes

No patients achieved CR or PR per irRECIST. Three patients (27%) had a best response of stable disease and 8 (73%) had PD. All 11 patients had a PFS event per irRECIST (death, *n* = 2; PD, *n* = 9). Median PFS per irRECIST was 3.7 months (95% CI, 1.7–5.4), with an irPFS rate at week 26 of 9%.

### Safety outcomes

No DLTs were observed. Treatment-related AEs were reported for 10 patients (91%; Table 2). The most common treatment-related AEs were diarrhea (55%), fatigue (45%), myalgia (36%), arthralgia (27%), chills (27%), nausea (27%), and pruritus (27%). Most treatment-related AEs were mild to moderate in severity. No grade 4 or 5 treatment-related AEs occurred. Two grade 3 treatment-related AEs were reported that were considered related to ipilimumab (diarrhea, *n* = 2 [18%]). No patients had grade 3 treatment-related AEs that were considered related to V937. Serious treatment-emergent AEs occurred in 4 patients (36%) and included grade 3 acute myocardial infarction, pyrexia, and hydronephrosis in 1 patient, grade 3 bile duct obstruction in 1 patient, grade 2 brain edema in 1 patient, and grade 2 colitis in 1 patient. Of these serious treatment-emergent AEs, colitis was considered probably related to ipilimumab; all other events were considered unrelated to either study treatment. Treatment with V937 and ipilimumab was delayed due to acute myocardial infarction, pyrexia, and hydronephrosis, and both study treatments were withheld during the time of colitis. Bile duct obstruction was ongoing at the time of the database cutoff date, while all other serious treatment-emergent AEs had resolved. Treatment-emergent AEs of diarrhea and noncardiac chest pain (*n* = 1 each) led to treatment discontinuation for 2 patients (18%). Five patients (45%) died during the study, but no deaths were considered related to study treatment.

**Table 2:** Treatment-related AEs

	Patients, n (%) *N* = 11		
	Related to V937	Related to ipilimumab	Overall
Patients with any treatment-related AE	9 (82)	10 (91)	10 (91)
Treatment-related AEs occurring in > 1 patient^a^			
Chills	3 (27)	1 (9)	3 (27)
Fatigue	3 (27)	4 (36)	5 (45)
Myalgia	3 (27)	3 (27)	4 (36)
Leukopenia	2 (18)	0	2 (18)
Nausea	2 (18)	2 (18)	3 (27)
Pruritus	2 (18)	3 (27)	3 (27)
Arthralgia	1 (9)	3 (27)	3 (27)
Diarrhea	1 (9)	5 (45)	6 (55)
Headache	1 (9)	1 (9)	2 (18)
Maculopapular rash	1 (9)	1 (9)	2 (18)

*AE* adverse event

^a^All treatment-related AEs were grade 1 or 2 with the exception of ipilimumab-related grade 3 diarrhea in 2 patients. No grade 4 or 5 treatment-related AEs were reported

## Discussion

Metastatic uveal melanoma is regarded as a difficult to treat tumor type (Chattopadhyay et al. 2016), since the disease is highly resistant to systemic chemotherapy (Chattopadhyay et al. 2016) and has shown minimal response to immunotherapy (Algazi et al. 2016; Maio et al. 2013; Najjar et al. 2020; Namikawa et al. 2020; Pelster et al. 2021; Rossi et al. 2019; Zimmer et al. 2015). Previous studies in patients with metastatic uveal melanoma have reported ORRs of 0%–7% with ipilimumab monotherapy (Maio et al. 2013; Wessely et al. 2020; Zimmer et al. 2015) and 12% with ipilimumab plus nivolumab (Najjar et al. 2020). In the CLEVER study, V937 was combined with ipilimumab based on the rationale that the combination of V937 and ipilimumab might result in augmented T-cell responses with consequent improved clinical activity. However, the combination regimen did not result in objective responses for any patients, although 3 patients did have stable disease as their best response.

Metastatic uveal melanomas are often immunologically “cold” tumors (Luke et al. 2020), except for a subset of patients (Rothermel et al. 2016). The mechanisms that contribute to these immunologic features are incompletely understood. A low tumor mutational burden has been suggested to contribute (Rossi et al. 2021; Rothermel et al. 2016). Additionally, the unique microenvironment of uveal melanomas may limit immune infiltration both within the eye and at sites of metastasis. The most common location of uveal melanoma metastases, the liver, displays known mechanisms of immune tolerance that may contribute to resistance to therapy (Tiegs and Lohse 2010). Nevertheless, the recent approval of tebentafusp suggests that immunotherapies do have activity in some metastatic uveal patients (US Food and Drug Administration 2022).

Oncolytic viruses carry risks such as the potential for triggering an antiviral immune response and off-target infection (Goradel et al. 2021). These risks are mitigated using viruses that are not associated with serious illness (Shafren et al. 2004). V937 is one such virus, associated with mild respiratory illness similar to rhinoviruses (Couch et al. 1970; Spickard et al. 1963; Supian et al. 2021; Zou et al. 2017). Although several treatment-related AEs were attributed to V937 (all grade 1 or 2), few patients experienced the flu-like symptoms that are commonly reported with V937 treatment (Supian et al. 2021; Zou et al. 2017). This finding may suggest that intravenous administration of the virus does not lead to extratumoral infection, thus supporting the feasibility of V937 as a systemic oncolytic therapy for patients with other cancer types, including cutaneous melanoma. The symptoms commonly associated with ipilimumab administration were mostly mild or moderate, but 2 grade 3 AEs of diarrhea were attributed to ipilimumab. No new or unexpected safety signals were reported.

Although the combination of intravenous V937 and ipilimumab was tolerable in patients with uveal melanoma and metastases to the liver, there was limited antitumor activity. A limitation of our study was that neutralizing antibodies against V937 and their potential effect on responses were not assessed. The CLEVER study demonstrates the feasibility of combining oncolytic virotherapy with immunotherapy and supports the safety profile of this particular therapeutic combination for patients with other cancers. The current study did not evaluate intratumoral V937 and ipilimumab. While intratumoral therapy for uveal melanoma may be challenging, it is feasible, and likely applicable, as most patients have metastasis to the liver (Carvajal et al. 2017). Translational studies to identify patients more likely to respond to this therapeutic combination may further aid in the potential application of such regimens across tumor types.

## Data Availability

Merck Sharp & Dohme LLC, a subsidiary of Merck & Co., Inc., Rahway, NJ, USA (MSD) is committed to providing qualified scientific researchers access to anonymized data and clinical study reports from the company’s clinical trials for the purpose of conducting legitimate scientific research. MSD is also obligated to protect the rights and privacy of trial participants and, as such, has a procedure in place for evaluating and fulfilling requests for sharing company clinical trial data with qualified external scientific researchers. The MSD data sharing website (available at: http://engagezone.msd.com/ds_documentation.php) outlines the process and requirements for submitting a data request. Applications will be promptly assessed for completeness and policy compliance. Feasible requests will be reviewed by a committee of MSD subject matter experts to assess the scientific validity of the request and the qualifications of the requestors. In line with data privacy legislation, submitters of approved requests must enter into a standard data-sharing agreement with MSD before data access is granted. Data will be made available for request after product approval in the US and EU or after product development is discontinued. There are circumstances that may prevent MSD from sharing requested data, including country or region-specific regulations. If the request is declined, it will be communicated to the investigator. Access to genetic or exploratory biomarker data requires a detailed, hypothesis-driven statistical analysis plan that is collaboratively developed by the requestor and MSD subject matter experts; after approval of the statistical analysis plan and execution of a data-sharing agreement, MSD will either perform the proposed analyses and share the results with the requestor or will construct biomarker covariates and add them to a file with clinical data that is uploaded to an analysis portal so that the requestor can perform the proposed analyses.
